# A data mining approach using cortical thickness for diagnosis and characterization of essential tremor

**DOI:** 10.1038/s41598-017-02122-3

**Published:** 2017-05-19

**Authors:** J. Ignacio Serrano, Juan P. Romero, Ma Dolores del Castillo, Eduardo Rocon, Elan D. Louis, Julián Benito-León

**Affiliations:** 1 Neural and Cognitive Engineering group, Automation and Robotics Center (CAR), CSIC-UPM, Arganda del Rey, Spain; 2 Faculty of Biosanitary Sciences, Francisco de Vitoria University, Pozuelo de Alarcón, Madrid, Spain; 3 Brain Damage Service, Hospital Beata Maria Ana, Madrid, Spain; 4 Department of Neurology, Yale School of Medicine, New Haven, CT USA; 5 Department of Chronic Disease Epidemiology, Yale School of Public Health, New Haven, CT USA; 6 Center for Neuroepidemiology and Clinical Neurological Research, Yale School of Medicine and Yale School of Public Health, New Haven, CT USA; 7 Department of Neurology, Center of Biomedical Network Research on Neurodegenerative Diseases (CIBERNED), University Hospital 12 de Octubre, Madrid, Spain; 8 Department of Medicine, Faculty of Medicine Complutense University, Madrid, Spain

## Abstract

Essential tremor (ET) is one of the most prevalent movement disorders. Being that it is a common disorder, its diagnosis is considered routine. However, misdiagnoses may occur regularly. Over the past decade, several studies have identified brain morphometric changes in ET, but these changes remain poorly understood. Here, we tested the informativeness of measuring cortical thickness for the purposes of ET diagnosis, applying feature selection and machine learning methods to a study sample of 18 patients with ET and 18 age- and sex-matched healthy control subjects. We found that cortical thickness features alone distinguished the two, ET from controls, with 81% diagnostic accuracy. More specifically, roughness (i.e., the standard deviation of cortical thickness) of the right inferior parietal and right fusiform areas was shown to play a key role in ET characterization. Moreover, these features allowed us to identify subgroups of ET patients as well as healthy subjects at risk for ET. Since treatment of tremors is disease specific, accurate and early diagnosis plays an important role in tremor management. Supporting the clinical diagnosis with novel computer approaches based on the objective evaluation of neuroimage data, like the one presented here, may represent a significant step in this direction.

## Introduction

Essential tremor (ET) is one of the most common movement disorders^1, 2^. Classically, it has been considered a benign and monosymptomatic disorder characterized primarily by kinetic arm tremor. However, an emerging view that is gaining wider support is that it may be a family of diseases unified by the presence of kinetic tremor, while also displaying etiological, pathologic, and clinical heterogeneity^3–5^. Recent evidence suggests that it is a family of disorders rather than a single condition, and that contrary to the traditional perspective, it may be a neurodegenerative disease^6^.

Aside from motor manifestations, ET is also associated with a number of non-motor manifestations, including depressive symptoms^7^, changes in sleep patterns^8^, and hearing impairment, among others^9^. In addition to non-motor features, some ET patients exhibit mild cognitive deficits, mainly in attention and frontal executive functions, verbal memory and visuospatial processes, which may be explained by frontal cortical or frontal cortical–cerebellar pathway dysfunction^10–14^.

As a highly prevalent neurological disorder, the diagnosis of ET is considered routine. However, previous work suggests that misdiagnoses is common^15^. Among other things, these misdiagnoses can lead to treatment errors. The diagnosis ET is over-applied even among experienced neurologists. For example, according to one study, approximately 1 in 3 patients who carried an “ET” diagnosis did not have ET; many of these had Parkinson’s disease and dystonia^15^. There is therefore a need to search for tools to better characterize and diagnose ET.

Magnetic resonance imaging (MRI) and radiotracer-based imaging techniques have proven to be helpful tools to enhance the accuracy of clinical diagnosis in movement disorders research^16^. Over the past decade, several studies have identified brain morphometric changes in ET^17, 18^, but these changes, including their causation, remain poorly understood.

Advances in brain morphometric techniques have enabled surface based approaches to more reliably quantify cortical structure. A surface-based approach offers several advantages over standard volumetric methods. Specifically, an assessment of cortical thickness provides a directly interpretable metric, allows for detection of sub-voxel changes^19^ while being less sensitive to inaccuracies of spatial normalization and smoothing^20^, and has been well validated^21^. Only two studies have assessed the pattern of cortical thickness in ET^22, 23^. Chung *et al*.^22^ analyzed the pattern of cortical thickness in 18 ET patients who had responded to propranolol and 14 who had not. Relative to responders, the non-responder group had more severe atrophy in the left orbitofrontal cortex and right temporal cortex^22^. In a voxel-based morphometry and cortical thickness study involving 14 ET patients, 12 dystonia patients, and 23 age- and sex-matched healthy control subjects, Cerasa *et al*.^23^ reported subtle atrophy of the anterior cerebellar cortex in the ET patients. However, we should keep in mind that the distribution of cortex thickness is not uniform by layer, neither is the variation in the thickness of the cortical layers proportional to the variation in the total thickness, nor is the location and progression of subtle cortical atrophy the same among individuals with the same neurodegenerative disease^24^. Hence, there is also a need for new more reliable variables to analyze the pattern of cortical thickness. “Roughness”, defined as the standard deviation of the thickness within a certain area above, may be a promising metric to overcome these limitations. To the authors’ knowledge, there is no work addressing the role of roughness in the structural and functional characterization of ET.

The application of multiple macroscopic and microscopic neuroimaging modalities, combined with personalized information relative to motor, cognitive and behavioral symptoms, could be the prerequisite for a comprehensive classification and correct diagnosis of ET. To this aim, feature selection methods and machine learning algorithms were applied for two main purposes: (1) contrasting the informative value of cortical thickness and roughness with respect to the volumetric features of the brain for diagnostic purposes in ET, and (2) finding the optimum subset of structural features that best characterize ET.

## Methods

### Ethical aspects

All the participants included in the study gave their written informed consent after full explanation of the procedure. The study, which was conducted in accordance with the principles of the Helsinki declaration of 1975, was approved by the ethical standards committee on human experimentation at the University Hospital “12 de Octubre” (Madrid). Written (signed) informed consent was obtained from all enrollees.

### Participants

ET patients were consecutively recruited from October 2012 to July 2013 from the outpatient neurology clinics of the University Hospital “12 de Octubre” in Madrid (Spain) after obtaining an appropriate informed consent. Patients with history of dementia, stroke, epilepsy, head injury or serious medical illness were excluded. Furthermore, based on a detailed clinical mental status examination, we excluded patients with Diagnostic and Statistical Manual of Mental Disorders (DSM)–IV criteria for dementia^25^.

Two neurologists with expertise in movement disorders (JPR and JB-L), who were blinded to the MRI results, examined the patients and used the Fahn-Tolosa-Marìn tremor rating scale to assign a total tremor score (range = 0–144)^26^. Diagnoses of ET were assigned by the 2 neurologists (JPR and JB-L) using the Consensus Statement on Tremor by the Movement Disorder Society^27^. Furthermore, all ET patients had a normal [(123) I]FP-CIT single photon emission computed tomography scan. All eligible ET patients underwent a detailed videotaped neurological examination. Each videotape was reviewed by a senior neurologist specializing in movement disorders (EDL) who re-assessed ET diagnosis using the Consensus Statement on Tremor by the Movement Disorder Society^27^. The ET patients were also followed at regular intervals (3 months, 6 months, or 12 months, based on clinical need) after the MRI procedure, and their clinical assessment, described above, was repeated. The mean duration of follow-up after the MRI procedure was 2.5 years (median = 2.7 years; range = 1.8–2.8 years).

Healthy controls were recruited either from relatives or friends of the health professionals working at the University Hospital “12 de Octubre” of Madrid (Spain) or among the relatives of patients who came to the neurological clinics for reasons other than ET (e.g., headache, dizziness). None reported having a first-degree or second-degree relative with ET. Each control was examined by two neurologists (JPR and JB-L), who were blinded to the MRI results, to further rule out any neurological or other serious conditions, including movement disorders, dementia, stroke, epilepsy, or head injury.

As this study was nested within the NEUROTREMOR project (http://www.neuralrehabilitation.org/projects/neurotremor/), a project whose main aim was to validate technically, functionally and clinically, a novel system for understanding, providing diagnostic support, and remotely managing tremors, most of the ET patients who were eligible refused to participate because of lack of time because the study would have required that they come to the hospital several times during the study for the performance of clinical, neurophysiological (magneto-electroencephalography and electromyography recordings), neuropsychological, and imaging evaluations. Given this constraint, of the 300 ET patients seen at outpatient neurology clinics of the University Hospital “12 de Octubre” in Madrid (Spain) from October 2012 to July 2013, only 47 were eligible for the study. Of these 47 ET patients who were eligible for the study, 19 had complete neuropsychological testing (see below) and an MRI procedure with cortical thickness data. Of these 19 ET patients, one was excluded from the final analyses because he developed incident Parkinson’s disease during follow-up. None of the patients and HCs were excluded because of neurological comorbidities or structural abnormalities on conventional MRI images.

The final sample of 18 ET patients did not differ to a significant degree from the 18 healthy controls in terms of age, sex, and educational level (Table 1). The mean tremor duration was 23.6 ± 16.6 years and the mean tremor rating scale score was 34.4 ± 15.5 (Table 1).

**Table 1:** Comparison of demographic and clinical characteristics of essential tremor patients vs. healthy controls.

	Healthy controls (N = 18)	Essential tremor patients (N = 18)	*p* value
Sex (men)	7 (38.9%)	10 (55.6%)	*χ*(*1*) = *1*.*001*, *p* = *0*.*317*
Age in years	63.3 ± 12.0	63.7 ± 10.5	*t*(*34*) = *−0*.*089*, *p* = *0*.*930*
Years of education	9.0 ± 3.3	7.8 ± 2.8	*t*(*34*) = *1*.*196*, *p* = *0*.*240*
Number of medications	1.8 ± 2.8	2.6 ± 2.4	*U* = *120*.*0*, *p* = *0*.*192*
Tremor severity^1^		34.4 ± 15.5	
Tremor duration in years		23.6 ± 16.6	
Presence of head tremor		5 (27.8%)	
Presence of voice tremor		3 (16.7%)	

Values are expressed as mean ± standard deviation. Student’s t test was used for parametric comparisons and Mann Whitney test for non parametric comparisons, and the chi-square test for sex proportion. ^1^Fahn–Tolosa–Marin Tremor Rating Scale.

### Cognitive assessment

All participants underwent a detailed neuropsychological evaluation that assessed the domains of attention, executive function, verbal memory, visual memory, visuospatial ability, and language. These tests have previously been described^28, 29^ and were chosen for the battery because they made minimal demands on motor processes, thereby avoiding effects of any hand tremor. Testing was performed by a trained neuropsychologist (V. P., see acknowledgments) using standardized procedures who was blinded to the clinical diagnosis as well as the MRI results. The neuropsychological examination was performed while taking their regular daily medication.

Attention and executive function were evaluated using a series of tests. First, participants underwent the Direct and Indirect Digit Span and the Coding-Digit Symbol subtests from the Wechsler Adult Intelligence Scale - Third Edition (WAIS-III) (higher scores indicate better cognitive performance)^30^. In the first, the examinee is required to repeat 3–9 digits forwards (direct) and backward (indirect)^30^. In the second, the numbers 1–7 have to be paired with symbols on a key presented to the examinee^30^. Second, the Similarities subtest from the WAIS-III was also administered^30^; in this test, which examines concrete, functional, and abstract concept formation, 19 items require the examinee to describe how two given things are alike^30^. Higher scores indicate better cognitive performance^30^. The Trail-making Test (TMT) is a measure of visuomotor coordination in which subjects must connect circles in one form (A) on the basis of a simple rule of consecutive numbers and in the second form (B) by alternating between numerical and alphabetical sequences^31^. For both forms, A and B, time for completion is the primary index of performance (lower scores indicate better cognitive performance). Third, the Stroop Color–Word Trial requires the participant to name the color of the ink in which a colored word is printed^32^. The task involves three test cards, one containing rows of colored rectangles, with the task being to name the colors as quickly as possible, one containing rows of color words (printed in black ink), with the task being to read the words as quickly as possible, and the third “interference” test consisting of rows of color words printed in ink colors incongruent with the word represented, with the task being to name the ink colors as quickly as possible^32^. The subject must ignore the word and name the color^32^. Fourth, the Wisconsin Card Sorting Test, a test of “set-shifting”, requires the examinee to discern the sort criterion of a set of cards based upon “correct” versus “incorrect” feedback given by the examiner^33^. The score for this test was the number of errors and perseverations (higher scores indicate worse performance)^33^. Fifth, the Tower of London was administered, a well-known test used for the assessment of executive function specifically to detect deficits in planning^34^. The test consists of two boards with pegs and several beads with different colors^34^. The examiner uses the beads and the boards to present the examinee with problem-solving tasks^34^. Finally, the Frontal Assessment Battery (FAB), a brief tool, designed to assess frontal lobe functions, including conceptualization, mental flexibility, motor programming, sensitivity to interference, inhibitory control, and environmental autonomy, was administered^35^.

To evaluate visuospatial ability, two tests were used. The first, the Benton Judgment of Line Orientation Test, is a standardized test of visuospatial skills, which measures a person’s ability to match the angle and orientation of lines in space^36^. The second, the Hooper Visual Organization Test^37^, is an instrument that measures visual organizational skills, and consists of line drawing of simple objects that have been cut into pieces and rearranged, such as in a puzzle. The examinee’s task is to name what the object would be if the pieces were put back together^37^. In both tests, higher scores indicate better cognitive function^36, 37^.

To evaluate verbal memory, we used the Wechsler Memory Scale-Third Edition (WMS-III) Word List^38^, which included four learning trials of 12 unrelated words. World List 1 is derived from the sum of the four trials^38^. A second list is then presented once for immediate recall, following which the examinee is asked to again recall the first list^38^. Free recall and recognition (yes-no format) of the initial words are later assessed after a delay interval^38^. Higher scores indicate better cognitive function^38^.

To evaluate visual memory, we used the Brief Visuospatial Memory Test-Revised^39^. In three learning trials, the examinee views the stimulus page and is asked to draw as many of the figures as possible^39^. A delayed recall trial is administered after a 25-minute delay^39^. Last, there is a recognition trial, in which the examinee is asked to identify which of 12 figures were included among the original ones^39^. Higher scores indicate better cognitive function^39^.

Language was evaluated using the following tests. First, the Boston Naming Test^40^, which assesses the ability to name pictures of objects through spontaneous responses and the need for various types of cueing (lower scores indicate greater cognitive impairment). Second, participants were asked to name as many items as possible from a semantic category (animals) (semantic fluency) (lower scores indicate greater cognitive impairment)^41^. Finally, the Controlled Oral Word Association Test (COWAT), a test that measures phonetic fluency, was administered^42^. Participants are provided three letters of the alphabet (F, A, and S), one letter at a time, and instructed to say as many words as possible that begin with this letter in a 60-second interval^42^. Higher scores indicate better cognitive performance^42^.

Depression was assessed with the 17-item version of the Hamilton Depression Rating Scale^43^. Higher scores reflect more depressive symptoms^43^.

Psychopathology and personality symptoms were assessed using the Personality Assessment Inventory (PAI), a widely used multidimensional 344-item self-report measure^44^. The PAI consists of 22 nonoverlapping scales: 4 validity scales, 11 clinical scales, 5 treatment consideration scales, and 2 interpersonal scales. For the present study, only clinical scales (somatic concerns, anxiety, anxiety related disorders, depression, mania, paranoia, schizophrenia, borderline features, antisocial features, alcohol problems, and drug problems) were used, and higher scores reflect greater psychopathology.

Seventy-three scores (total and partial scores) were obtained for each participant. Table 2 shows the mean and standard deviation scores of the tests that differed to a statistically significant degree between ET patients and controls.

**Table 2:** Cognitive and neuropsychiatric domains that were significantly different between essential tremor patients and healthy controls.

Cognitive domains	Healthy controls (N = 18)	Essential tremor patients (N = 18)	Student’s t test or Mann-Whitney U test, Bonferroni corrected
*Attention and Executive funtion*			
Trial Making Test-A, time for completion	52.7 ± 28.3	93.7 ± 53.5	*U* = *245*.*5*, *p* = *0*.*007*
Tower of London Tower, initiation time	51.3 ± 16.7	79.9 ± 29.2	*t*(*34*) = *−3*.*554*, *p* = *0*.*001*
Coding-Digit Symbol subtest from the WAIS-III, total score	49.9 ± 19.1	31.4 ± 17.5	*U* = *72*.*0*, *p* = *0*.*004*
Frontal Assessment Battery, fluency score	2.7 ± 0.6	2.0 ± 1.0	*U* = *98*.*0*, *p* = *0*.*044*
Frontal Assessment Battery, total score	16.7 ± 1.1	14.7 ± 3.3	*U* = *97*.*5*, *p* = *0*.*040*
*Language*			
Boston Naming Test, naming score	51.9 ± 5.5	42.3 ± 12.6	*t*(*34*) = *2*.*949*, *p* = *0*.*006*
Boston Naming Test, total score	52.5 ± 5.3	43.0 ± 12.8	*t*(*34*) = *2*.*916*, *p* = *0*.*006*
Controlled Oral Word Association Test, letter A	12.5 ± 6.4	7.6 ± 5.0	*U* = *88*.*5*, *p* = *0*.*019*
Controlled Oral Word Association Test, total score	35.8 ± 13.9	25.6 ± 13.5	*U* = *91*.*0*, *p* = *0*.*024*
*Visuospatial ability*			
Hooper Visual Organization Test, total score	39.0 ± 8.5	32.9 ± 9.2	*t*(*34*) = *2*.*054*, *p* = *0*.*048*
*Psychopathology & Personality*			
Personality Assessment Inventory, anxiety score	6.4 ± 4.4	10.5 ± 6.1	*t*(*34*) = *−2*.*185*, *p* = *0*.*037*
Personality Assessment Inventory, depression score	5.7 ± 3.9	10.5 ± 5.9	*t*(*34*) = *−2*.*765*, *p* = *0*.*010*

Values are expressed as mean ± standard deviation. Student’s t test was used for parametric comparisons and Mann Whitney test for non parametric comparisons.

The results of neuropsychological testing are shown in Table 2. In several domains, ET patients’ cognitive performance was significantly worse than that of the healthy controls. These differences involved selected tests of attention, executive function, language, visuospatial ability, and psychopathology and personality.

### Neuroimage acquisition

As there is no evidence that long-term anti-tremor medications influence cortical thickness in patients with ET, and the biological plausibility for such an effect is very low, ET patients continued taking medication for their disease, propranolol and/or primidone, during the MRI procedures.

Both patients and healthy controls were immobilized with a custom-fit blue bag vacuum mold (Medical Intelligence, Inc.) to prevent image artifacts. A strict criterion for head movement assessment was adopted (maximal absolute head movement less than 1.0  mm and 1.0° in the x, y, and z directions). Neither patients nor healthy controls were excluded from the analysis due to this criterion.

MRI data were acquired on each patient and control using a GE Signa 3.0 T scanner (General Electric Medical Systems, Milwaukee, WI) with a standard quadrature birdcage headcoil, using an axial 3D T1-weighted inversion-recovery fast gradient echo sequence (TR = 5.0 ms; TE = 2.2 ms; Flip Angle = 12°; TI = 750 ms; NEX = 1.0). A total of 176 contiguous 1-mm slices were acquired with a 240 × 240 matrix with an in-plane resolution of 1 × 1 mm, resulting in isotropic voxels. Standard sequences of the MRI scans were checked before inclusion of a patient or control. Those with structural abnormalities in the brain, affecting gray or white matter, were excluded prior to the image analysis. MRI studies and imaging processing were performed by a neuroradiologist (JA-L) and a physicist (JAH-T) who were blinded to the clinical diagnosis.

### Neuroimage processing

MRI images were processed to extract two types of information: volumetric features and cortical thickness features. Cortical reconstruction and volumetric segmentation was performed with the Freesurfer image analysis suite, which is documented and freely available for download online (http://surfer.nmr.mgh.harvard.edu/). Briefly, this processing includes motion correction and averaging^45^ of multiple volumetric T1 weighted images (when more than one is available), removal of non-brain tissue using a hybrid watershed/surface deformation procedure^46^, automated Talairach transformation, segmentation of the subcortical white matter and deep gray matter volumetric structures (including hippocampus, amygdala, caudate, putamen, ventricles)^47, 48^, intensity normalization^49^, tessellation of the gray matter white matter boundary, automated topology correction^50^, and surface deformation following intensity gradients to optimally place the gray/white and gray/cerebrospinal fluid borders at the location where the greatest shift in intensity defines the transition to the other tissue class^24^. Once the cortical models are complete, a number of deformable procedures can be performed for further data processing and analysis; these include surface inflation^51^, registration to a spherical atlas which utilizes individual cortical folding patterns to match cortical geometry across subjects^52^, fragmentation of the cerebral cortex into units based on gyral and sulcal structure^53^, and creation of a variety of surface based data including maps of curvature and sulcal depth. This method uses both intensity and continuity information from the entire three dimensional MR volume in segmentation and deformation procedures to produce representations of cortical thickness, calculated as the closest distance from the gray/white boundary to the gray/CSF (Cerebrospinal Fluid) boundary at each vertex on the tessellated surface^24^. The maps are created using spatial intensity gradients across tissue classes and are therefore not simply reliant on absolute signal intensity. The maps produced are not restricted to the voxel resolution of the original data, and thus are capable of detecting submillimeter differences between groups. The cortical thickness features are average values for each region. Additionally, for each cortical region, the standard deviation of the cortical thickness was also calculated as a measure of roughness.

Anatomical localization of the cerebral areas of altered white and gray matter was performed using the Talairach Daemon (www.talairach.org)^54^ after converting Montreal Neurological Institute coordinates into Talairach coordinates, by means of “ni2tal.m” Matlab script by Matthew Brett (http://www.mrc-cbu.cam.ac.uk/Imaging/Common).

The above processing steps yielded 129 white matter and grey matter volumetric features of the whole brain (except for the cerebellum) and 152 cortical thickness features (average plus roughness, i.e. standard deviation of the thickness), according to the Desikan-Killiany atlas, resulting in a total of 281 structural features from each subject. Table 3 shows the subset of structural features, from the 281 extracted, that revealed statistically significant differences between the two groups of participants. Among the differences presented in Table 3, only two are volumetric features. From those, only one refers to a subcortical structure - the left hippocampus. Therefore, most differences between the two groups were in terms of cortical thickness and roughness. The decreased thickness in ET patients in superior frontal and precentral areas (both left and right) could account for the motor symptoms of ET^17^. The dimished left hippocampus and left rostral anterior cingulate thickness in ET patients might explain increased anxiety and depressive symptoms, which have been described^7, 55, 56^. The remainder of frontal, temporal and cingulate differences could relate to differences in cognitive domains between the two groups (see Table 2).

**Table 3:** Average ± standard deviation for the structural features extracted from MRI that showed statistically significant differences between healthy controls and ET patients.

Features	Healthy controls (N = 18)	Essential tremor patients (N = 18)	Student’s t test or Mann-Whitney U test, Bonferroni corrected
lh-hippocampus-volume/ICV	0.0026 ± 0.0005	0.0023 ± 0.0003	*t*(*34*) = *2*.*185*, *p* = *0*.*036*
lh-entorhinal-volume	2072 ± 339	1762 ± 523	*t*(*34*) = *2*.*113*, *p* = *0*.*042*
lh-superiorfrontal-thickness	2.6867 ± 0.1166	2.5888 ± 0.1429	*t*(*34*) = *2*.*253*, *p* = *0*.*031*
lh-lateralorbitofrontal-thickness	2.6403 ± 0.1120	2.5516 ± 0.1264	*t*(*34*) = *2*.*229*, *p* = *0*.*033*
lh-precentral-thickness	2.5486 ± 0.1114	2.4398 ± 0.1394	*t*(*34*) = *2*.*585*, *p* = *0*.*014*
lh-supramarginal-thickness	2.5972 ± 0.1389	2.5018 ± 0.1396	*t*(*34*) = *2*.*054*, *p* = *0*.*048*
lh-temporal lobe-thickness	2.9744 ± 0.1273	2.8529 ± 0.1711	*t*(*34*) = *2*.*418*, *p* = *0*.*021*
lh-superior temporal-thickness	2.8380 ± 0.1419	2.7027 ± 0.2043	*t*(*34*) = *2*.*307*, *p* = *0*.*027*
lh-temporal pole-thickness	3.8554 ± 0.3878	3.6003 ± 0.3466	*t*(*34*) = *2*.*081*, *p* = *0*.*045*
lh-rostral anterior cingulate-thickness	2.9302 ± 0.2321	2.7606 ± 0.2121	*U* = *85*.*5*, *p* = *0*.*014*
lh-posterior temporal-roughness	0.4572 ± 0.0601	0.5017 ± 0.0697	*t*(*34*) = *−2*.*049*, *p* = *0*.*048*
lh-temporal pole-roughness	0.7118 ± 0.1529	0.8158 ± 0.0990	*t*(*34*) = *−2*.*420*, *p* = *0*.*021*
lh-posterior cingulate-roughness	0.5903 ± 0.0793	0.6474 ± 0.0865	*t*(*34*) = *−2*.*067*, *p* = *0*.*046*
rh-superiorfrontal-thickness	2.6792 ± 0.1192	2.5441 ± 0.1372	*t*(*34*) = *2*.*455*, *p* = *0*.*019*
rh-precentral-thickness	2.5302 ± 0.1072	2.4322 ± 0.1447	*t*(*34*) = *2*.*308*, *p* = *0*.*027*
rh-temporal lobe-thickness	3.0075 ± 0.1223	2.9078 ± 0.1568	*t*(*34*) = *2*.*128*, *p* = *0*.*041*
rh-superior temporal-thickness	2.8262 ± 0.1460	2.7007 ± 0.1932	*t*(*34*) = *2*.*198*, *p* = *0*.*035*
rh-temporal pole-thickness	3.9693 ± 0.2767	3.7626 ± 0.3186	*t*(*34*) = *2*.*078*, *p* = *0*.*045*
rh-parahippocampal-thickness	2.8016 ± 0.2811	2.6067 ± 0.2033	*t*(*34*) = *2*.*383*, *p* = *0*.*023*
rh-entorhinal-roughness	0.7413 ± 0.1244	0.8305 ± 0.1212	*t*(*34*) = *−2*.*180*, *p* = *0*.*036*

Values are expressed as mean ± standard deviation. Student’s t test was used for parametric comparisons and Mann Whitney test for non parametric comparisons. lh: left hemisphere; rh: right hemisphere; ICV: Intracranial volume.

### Feature selection

Each of the 281 features was scored according to the following well-known information measures:

- χ^2^ statistic. The chi-square statistic is a nonparametric statistical technique used to determine if a distribution of observed frequencies differs from the theoretical expected frequencies. Chi-square statistics use nominal data, thus instead of using means and variances, this test uses frequencies. The value of the chi-square statistic is given by
  1 $${\chi }^{2}=\sigma (\frac{(O-E)\times 2}{E})$$
  where χ^2^ is the chi-square statistic, *O* is the observed frequency and *E* is the expected frequency. Generally the chi-squared statistic summarizes the discrepancies between the expected number of times each outcome occurs (assuming that the model is true) and the observed number of times each outcome occurs, by summing the squares of the discrepancies, normalized by the expected numbers, over all the categories.
  - Rule accuracy. This measure represents the accuracy achieved by a single rule classifier only using the scored feature.
  - Information gain. This measure quantifies the difference between the amount of information required for classification before and after a splitting of the examples by the values of a single feature.
  - Gain ratio. This measure is derived from the previous one. It is the result of dividing the information gain by the intrinsic value of the class. Unlike Information gain, the Gain ratio biases against considering attributes with a large number of distinct values.
  - Gini-index. Gini-index is a measure of inequality designed to be applied to financial income. In feature selection, Gini-index is used to measure the purity of the clusters created by using a feature. It is closely related to the Area Under Curve (AUC).
  - Uncertainty. This measures the symmetrical uncertainty with respect to the class (control or ET). The higher this value of a feature, the more relevant it is considered. The relevance is given by the expression

2 $$Relevance(class,feature)=\,\frac{2\times (P(class)-P(class|\,feature))}{P(class)}+P(feature)$$

indicating *P* probability.

The six information measures were normalized between 0 and 1, the latter indicating the most discriminative power with respect to the ET condition. Since the information measures are sensitive to the type and size of data, the average score among the six values was calculated for each feature. Then, the features were ranked in descending order according to their average score.

### Automated classification

Features were grouped into four subsets: volume features, thickness features, roughness features (thickness standard deviation), and the total set of 281 features. Within each subset, features were ranked in descending order by the average information score. In turn, each of the four subsets of features was split in overlapping fragments of the first n% features of the ranked subset, ranging from 5% to 100%, in incrementing intervals of 2%. Five machine learning classifiers were applied to each of the feature subset fragments. The classifiers applied were^39^:

- Naive Bayes (NB) with Laplace correction.
- Support Vector Machine (SMV) of type C-SVC, radial basis as kernel function and epsilon equal to 0.001.
- Rule induction, with pureness of 0.99 and information gain as feature aggregation criterion.
- K-Nearest Neighbor (k-NN) with a weighted vote of the 3 nearest neighbors and Manhattan distance as similarity measure.
- Artificial Neural Network (ANN). Multi-layer perceptron with one hidden layer of 20 nodes, 10000 training cycles, learning rate of 0.2 and momentum equal to 0.15.

The remaining classifiers’ parameters were settled to default values, according to Rapid Miner Studio 5 tool (www.rapidminer.com). Classification was performed following a 10-cross-fold validation methodology. According to this methodology, the whole data set was split into 10 equally-sized parts. Then, one part was chosen as a validation set and the remaining nine parts were used as a learning set. The process was repeated ten times varying the part chosen for validation. The ultimate classification result was the average of the 10 validation sets. Grand-average classification accuracy was computed for each combination of *subset* × *classifier* × *fragment*.

#### Statistical analyses for demographic, clinical, and cognitive domains of ET patients vs. healthy controls

Statistical analyses for demographic, clinical and cognitive domains of ET patients vs. healthy controls were performed in SPSS Version 23.0 (IBM Corp., NY, USA). All tests were two sided, and significance was accepted at the 5% level (alpha = 0.05). Comparison means of groups was made by t-test for normally distributed data and by Mann-Whitney test for non-normally distributed data, where appropriate. For pairwise comparison among multiple groups Bonferroni posthoc test was used. The χ2 test was used to analyze differences in categorical variables.

## Results

**Figure 1:**
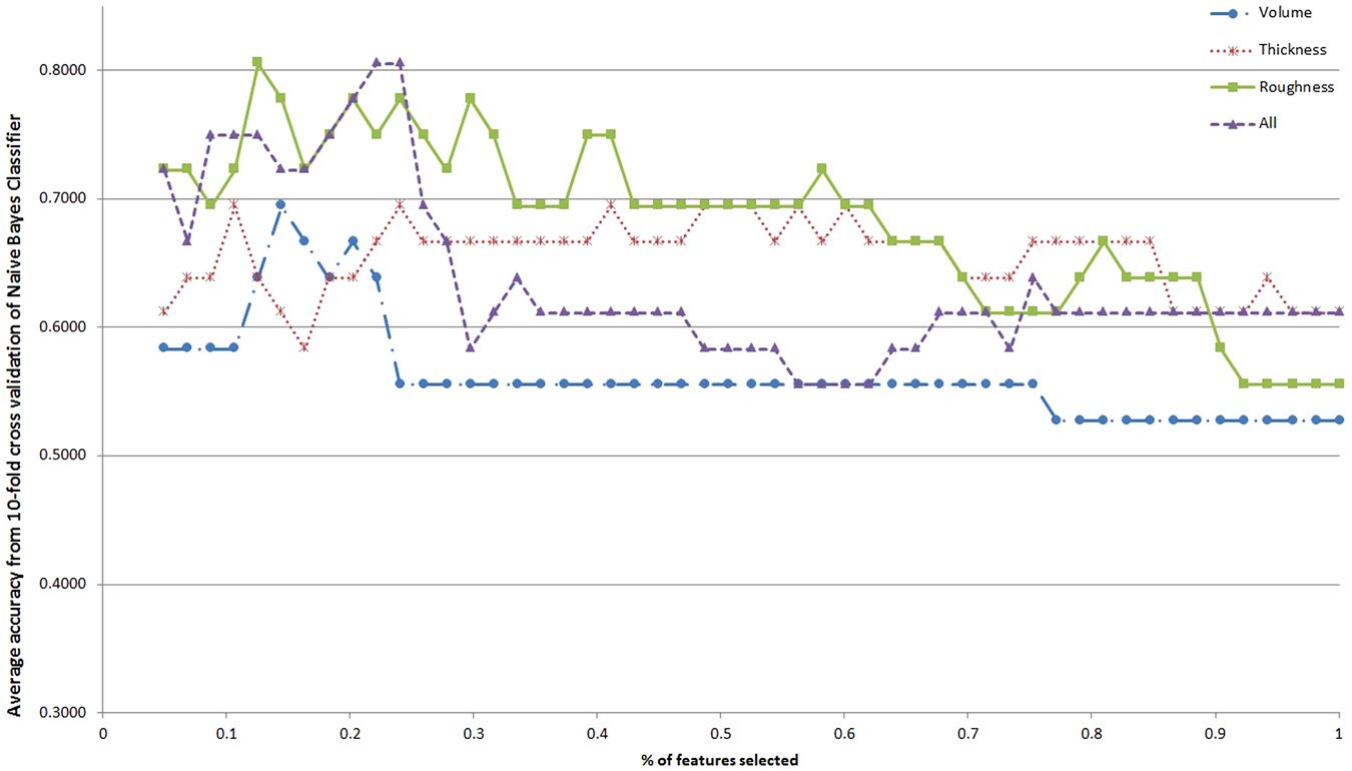
Classification accuracy with respect to the percentage of the feature ranking taken for the different feature types with Naive Bayes classifier.

**Figure 2:**
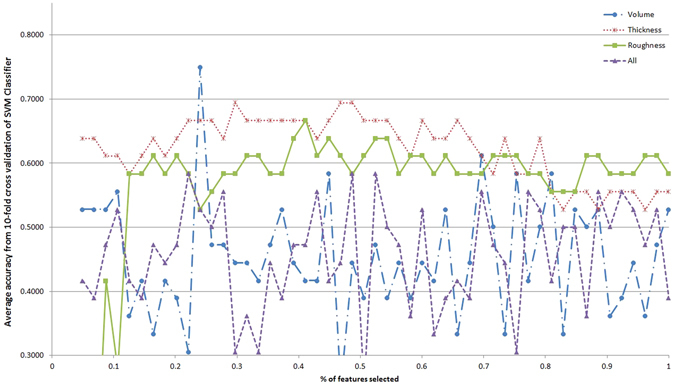
Classification accuracy with respect to the percentage of the feature ranking taken for the different feature types with SVM classifier.

**Figure 3:**
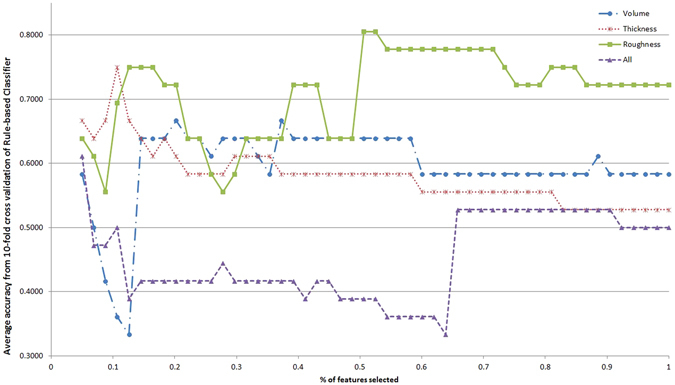
Classification accuracy with respect to the percentage of the feature ranking taken for the different feature types with Rule-based classifier.

**Figure 4:**
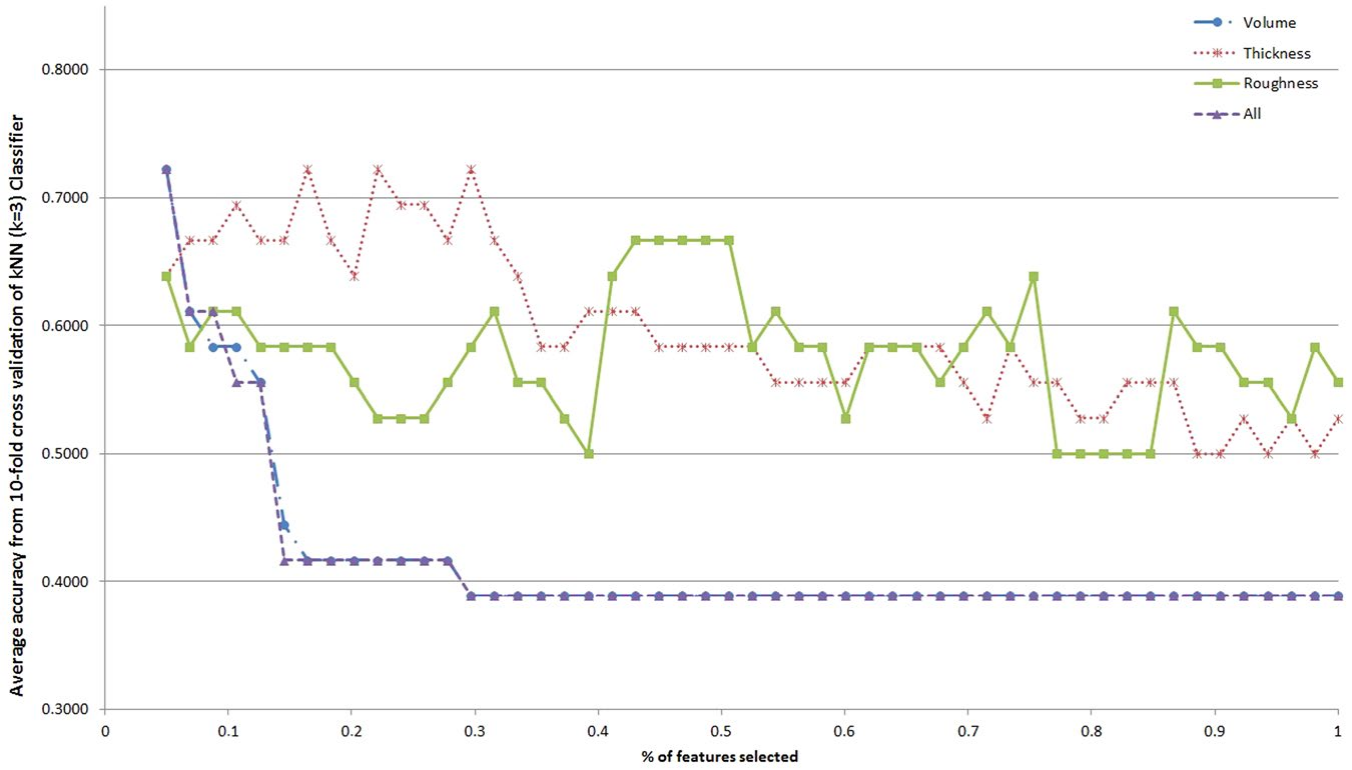
Classification accuracy with respect to the percentage of the feature ranking taken for the different feature types with kNN classifier.

**Figure 5:**
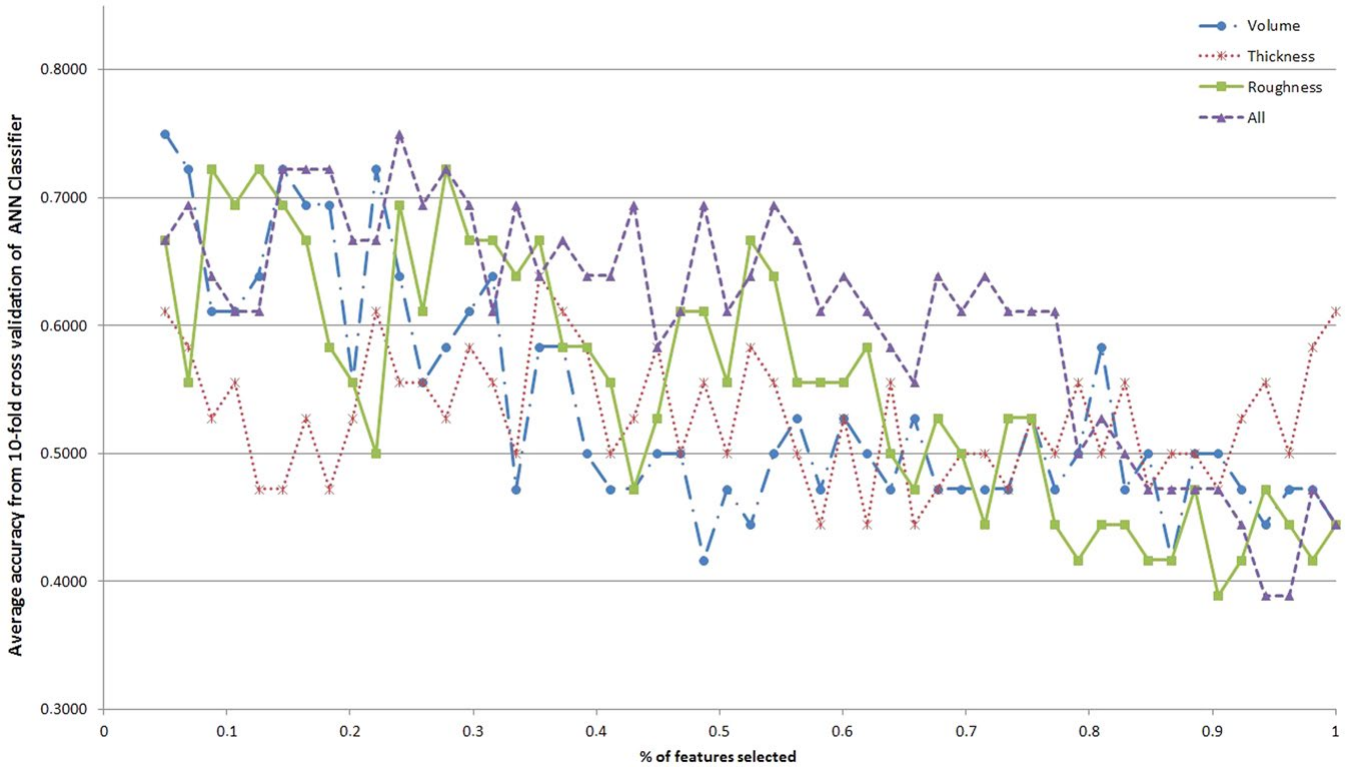
Classification accuracy with respect to the percentage of the feature ranking taken for the different feature types with Neural Network classifier.

Although some single features might have good discriminative power, it is the combination of such features that can uniquely characterize ET. In this sense, automated classification is a suitable approach to obtain the best subset of features. Furthermore, the classifiers are easily translatable to decision support systems. Figures 1–5 show the classification accuracy of the five different classifiers described, for each type of feature in each line and increasing percentages of the total feature ranking considered in the x-axis. Accuracy value indicates the percentage of participants correctly classified. It is evident that the results vary depending on the classifier, the feature type and the number of features taken. Generally, accuracy decreases as the number of features increases. This is a typical effect in automated classification, due to the noise introduced by less informative features.

Table 4 shows the average accuracy on all feature subset fragments for the three types of features and the five classifiers. Thickness features obtained the best average accuracy with SVM and kNN classifiers, while roughness features yield the best average accuracy with NB, and Rule induction classifiers, both presenting statistically significant differences with volume features and the three types together. Roughness features also obtained the highest average accuracy with the Rule induction classifier. With the ANN classifier, the best accuracy was achieved by the three feature types together. However, the grand average accuracy values did not present statistically significant differences among the feature types (Student’s t-test, Bonferroni corrected).

**Table 4:** Average classification accuracy for the different combinations of classifiers and types of features among all feature set sizes.

Classifier	All	Thickness	Roughness	Volume
Naive Bayes	0.6356_*a*_ ± 0.0664	0.6547_*a*,*b*_ ± 0.0285	0.6819_*b*_ ± 0.0653	0.5627_*c*_ ± 0.0388
Support Vector Machine	0.4570_*a*_ ± 0.0823	0.6231_*b*_ ± .0.0485	0.5648_*c*_ ± 0.1208	0.4537_*a*_ ± 0.0899
Rule	0.4532_*a*_ ± 0.0661	0.5806_*b*_ ± 0.0452	0.7092_*c*_ ± 0.0661	0.5948_*b*_ ± 0.0665
K-Nearest Neighbor	0.4150_*a*_ ± 0.0688	0.5959_*b*_ ± 0.0634	0.5773_*b*_ ± 0.0476	0.4156_*a*_ ± 0.0687
Artificial Neural Network	0.6067_*a*_ ± 0.0922	0.5294_*b*_ ± 0.0486	0.5534_*b*_ ± 0.0979	0.5365_*b*_ ± 0.0878
Average	0.5135_*a*_ ± 0.1002	0.5967_*a*_ ± 0.0470	0.6173_*a*_ ± 0.0726	0.5127_*a*_ ± 0.0754

Accuracy range is between 0 and 1. Numbers with no shared subindex (a, b, c) presented statistically significant difference (p < 0.05, Student’s t-test, Bonferroni-corrected).

Finally, Table 5 presents the overall highest accuracy values, together with the number of features used, for each feature type and classifier. Overall, the information provided by roughness features is the most discriminative, obtaining the best average maximum results for all classifiers with a relative low number of features. Again, the maximum accuracy values did not present significant differences among feature types (Student’s t-test, Bonferroni corrected).

**Table 5:** Maximum classification accuracy values for the different combinations of classifiers and types of features.

	Volume		Thickness		Roughness		All	
	Accuracy	Number of features used	Accuracy	Number of features used	Accuracy	Number of features used	Accuracy	Number of features used
Naive Bayes	0.6944	19	0.6944	37	0.8056	10	0.8056	62
Support Vector Machine	0.7500	31	0.6944	23	0.6667	36	0.5833	62
Rule	0.6667	26	0.7500	8	0.8056	36	0.6111	14
K-Nearest Neighbor	0.7222	6	0.7222	17	0.6667	33	0.7222	14
Artificial Neural Network	0.7500	6	0.6389	28	0.7222	10	0.7500	67
**Average**	*0*.*7167*	*17*.*60*	*0*.*70*	*22*.*60*	*0*.*7333*	*25*.*00*	*0*.*6944*	*43*.*80*
**Standard deviation**	*0*.*0362*	*11*.*4149*	*0*.*0412*	*10*.*9681*	*0*.*0697*	*13*.*7477*	*0*.*0942*	*27*.*280*

Accuracy range is between 0 and 1. There was no significant difference among average maximum values (*p* < 0.05, Student’s t-test, Bonferroni-corrected).

Given that the rule-based classifier produced the most easily interpretable output and it presented the best accuracy (~81% with roughness features, Table 5), it was worth studying the rule model obtained by this approach. Contrary to the classification models, the model presented next (rules 1 to 5) is obtained from the whole data set (no training/test split). Therefore, it is rather descriptive. The number of different subjects covered by each rule is showed as (Control/Essential Tremor) at the end of each rule:

**if** *rh Inferior parietal roughness* ≤ 0.666 **and** *lh lateral occipital roughness* ≤ 0.609 **then** *HEALTHY* (9/0)      (1)

**else if** *rh fusiform roughness* ≤ 0.696 **then** *ET* (0/12)           (2)

**else if** *lh medial orbitofrontal roughness* ≤ 0.841 **and** *lh frontal lobe roughness* > 0.558 **then** *HEALTHY* (8/0) (3)

**else if** *lh pars triangularis roughness* > 0.514 then *ET* (0/6)      (4)

**else** *HEALTHY* (1/0)              (5)

where ‘*lh*’ and ‘*rh*’ denote left-hemisphere and right-hemisphere, respectively.

To assess the possible correlations of these groups with cognitive function, Table 6 presents the scores from the neuropsychological tests performed that presented statistically significant differences among the groups defined by the rules. While all of the tests delineated in the Methods section were included in this analysis, this table includes only those that demonstrated statistically significant differences among any pair of rule-based groups.

**Table 6:** Demographic, clinical characteristics, and neuropsychological that presented statistically significant differences between healthy controls and ET patients, covered by rules 1 to 5.

	Control-Rule 1 (N = 9)	ET-Rule 2 (N = 12)	Control-Rule 3 (N = 8)	ET-Rule 4 (N = 6)	Control-Rule 5 (N = 1)
***Age***	57.6_a_ ± 12.2	61.8_a_ ± 12.3	67.9_a_ ± 8.7	67.3_a_ ± 4.5	79.0
***Sex*** (***1:male; 2:female***)	1.44_a_ ± 0.53	1.50_a_ ± 0.52	1.88_a_ ± 0.35	1.33_a_ ± 0.52	1.0
***Years of education***	11.0_a_ ± 2.2	8.3_a,b_ ± 2.5	7.0_b_ ± 3.2	6.8_b,c_ ± 3.4	8.0
***Cognitive domains***					
*Attention & Executive funtion*					
Trial Making Test-A, time for completion	35.6_a_ ± 10.7	79.4_a,b_ ± 43.4	74.7_a,b_ ± 28.4	122.3_b_ ± 64.1	30.0
Tower of London Tower, initiation time	50.8_a_ ± 20.5	84.9_b_ ± 30.0	50.4_a_ ± 13.2	69.0_a,b_ ± 27.1	64.0
Coding-Digit Symbol subtest from the Wechsler Adult Intelligence Scale - Third Edition, total score	63.1_a_ ± 12.0	34.6_b_ ± 20.5	35.2_b_ ± 16.0	25.0_b_ ± 6.5	49.0
*Language*					
Boston Naming Test, naming score	55.0_a_ ± 3.2	41.8_b_ ± 14.6	49.0_a,b_ ± 6.1	43.3_a,b_ ± 8.2	47.0
Boston Naming Test, total score	55.6_a_ ± 2.9	42.5_b_ ± 14.7	49.7_a,b_ ± 5.8	44.0_a,b_ ± 8.8	47.0
Controlled Oral Word Association Test, letter A	15.6_a_ ± 6.2	8.7_b_ ± 5.7	9.2_a,b_ ± 5.5	5.17_b_ ± 2.5	11.0
Controlled Oral Word Association Test, total score	42.3_a_ ± 14.2	27.7_a,b_ ± 16.0	28.1_a,b_ ± 10.9	21.50_b_ ± 5.2	38.0
*Visuospatial ability*					
Hooper Visual Organization Test, total score	44.0_a_ ± 8.9	34.7_a,b_ ± 10.3	33.5_a,b_ ± 4.2	29.5_b_ ± 5.61	38.0
*Memory*					
Wechsler Memory Scale-Third Edition, Word List 1	5.4_a_ ± 1.5	4.2_a,b_ ± 1.5	3.4_b_ ± 1.4	4.17_a,b_ ± 1.2	5.0
Brief Visuospatial Memory Test-Revised, recognition trial	11.1_a_ ± 1.7	8.6_a,b_ ± 2.8	8.6_a,b_ ± 2.8	6.6_b_ ± 4.4	10.0
***Psychopathology & Personality***					
Personality Assessment Inventory, anxiety score	5.7_a_ ± 4.0	12.2_b_ ± 6.2	8.5_a,b_ ± 4.6	6.4_a,b_ ± 3.8	1.0
Personality Assessment Inventory, depression score	4.6_a_ ± 3.4	11.4_b_ ± 6.5	8.3_a,b_ ± 3.3	8.4_a,b_ ± 3.8	0.0

Numbers with no shared subindex (a, b, c) presented statistically significant difference (p < 0.05, Student’s t-test, Bonferroni-corrected).

Structurally, Table 7 shows the features that presented statistically significant differences between the groups defined by the rules.

**Table 7:** Average and standard deviation measures of the cortical-related features that presented statistically significant differences between groups covered by each rule (1 to 5).

**Feature**	Control-Rule 1 (N = 9)	ET-Rule 2 (N = 12)	Control-Rule 3 (N = 8)	ET-Rule 4 (N = 6)	Control-Rule 5 (N = 1)
lh precentral thickness	2.5339_a,b_ ± 0.1192	2.4083_a_ ± 0.1451	2.5894_b_ ± 0.0796	2.5030_a, b_ ± 0.1122	2.35401
lh superior parietal roughness	0.5593_a_ ± 0.0309	0.6218_a, b_ ± 0.0785	0.6509_b_ ± 0.0487	0.5993_a, b_ ± 0.0491	0.55301
lh InferiorTemporal roughness	0.7504_a_ ± 0.0435	0.7847_a, b_ ± 0.0587	0.8271_b_ ± 0.0502	0.7550_a, b_ ± 0.0401	0.78201
rh parietal lobe roughness	0.6041_a_ ± 0.0348	0.6497_a, b_ ± 0.0447	0.6626_b_ ± 0.0292	0.6291_a, b_ ± 0.0414	0.62601
rh Inferior parietal roughness	0.6150_a_ ± 0.0440	0.720_b_ ± 0.0510	0.7460_b_ ± 0.0670	0.7060_b_ ± 0.0820	0.62310
rh supramarginal roughness	0.6081_a_ ± 0.0469	0.6547_a, b_ ± 0.0556	0.6910_b_ ± 0.0617	0.6328_a, b_ ± 0.0664	0.64001
rh middle temporal roughness	0.7032_a_ ± 0.0625	0.7312_a, b_ ± 0.0502	0.7925_b_ ± 0.0653	0.7735_a, b_ ± 0.0642	0.83501
rh fusiform roughness	0.6902_a,b_ ± 0.0478	0.6429_a_ ± 0.0427	0.7336_b, c_ ± 0.0429	0.7653_c_ ± 0.0560	0.76101

Numbers with no shared subindex (a, b, c) presented statistically significant difference (p < 0.05, Student’s t-test, Bonferroni-corrected). lh: left hemisphere; rh: right hemisphere.

**Figure 6:**
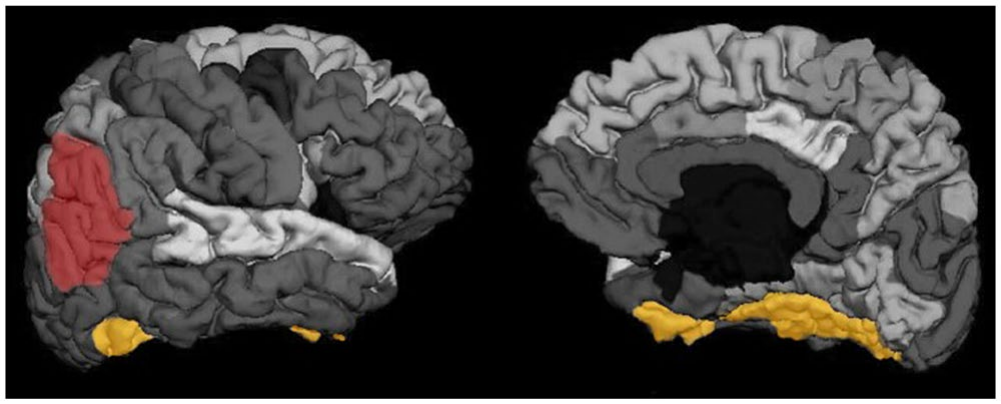
Highlighted cortical areas according to the Desikan-Killiany Atlas whose roughness difference distinguished between control and ET conditions. Yellow area’s roughness also differenciated the two ET groups derived from the rule model. Left: lateral surface of the rigth hemisphere; Right: medial surface of the right hemisphere.

Table 8 summarizes the cognitive and structural differences between each pair of groups of subjects defined by the rules, which presented statistical significance. The rule model showed that ET patients were distinguished from healthy controls in two different groups (rules 2 and 4). This division is interesting because it might be pointing to clinical subtypes of ET. The structural areas, whose roughness significantly differentiated conditions and the two ET groups, are depicted in Fig. 6 according to the Desikan-Killiany Atlas used.

**Table 8:** Neuropsychological tests and cortical-related features that presented statistically significant difference between each pair of groups defined by the rules, and that were not significantly correlated with educational level between pairs of groups.

	Control-Rule 3	ET-Rule 2	ET-Rule 4
Control-Rule 1	Years of education (>) Wechsler Memory Scale-Third Edition, Word List 1 (>) Coding-Digit Symbol subtest from the WAIS-III, total score (>) lh superior parietal roughness (<)	Tower of London Tower, initiation time (<) Coding-Digit Symbol subtest from the WAIS-III, total score (>) Boston Naming Test, naming score (>) Boston Naming Test, total (>) Controlled Oral Word Association Test, letter A (>) Personality Assessment Inventory, anxiety (<) Personality Assessment Inventory, depression (<) rh inferior parietal roughness (<)	Years of education (>) Brief Visuospatial Memory Test-Revised, recognition trial (>) Trial Making Test-A, time for completion (<) Coding-Digit Symbol subtest from the WAIS-III, total score (>) Hooper Visual Organization Test, total score (>) Controlled Oral Word Association Test, letter A (>) Controlled Oral Word Association Test, total score (>) rh inferior parietal roughness (<)
Control-Rule 3	lh inferior temporal roughness (<) rh parietal lobe roughness (<) rh inferior parietal roughness (<) rh supramarginal roughness (<) rh middle temporal roughness (<)	Tower of London Tower, initiation time (<) lh precentral thickness (>) rh fusiform roughness (>)	rh fusiform roughness (<)
ET-Rule 2			rh fusiform roughness (<)

Statistically significant difference at *p* < 0.05 (Student’s t−test, Bonferroni−corrected). lh: left hemisphere; rh: right hemisphere. “<”: group in row < group in colum; “>”:group in row > group in colum.

According to Table 8, the two groups of ET defined by the rule model only presented significant differences in the roughness of the right fusiform cortex (Brodmann areas 20 and 37). Since there were no significant differences between the two ET groups in neuropsychological terms (data not shown), the structural difference might account for clinical subtyping. However, no significant difference regarding disease duration or disease severity was found between the two groups, as shown in Table 9.

**Table 9:** Clinical variables of the ET groups defined by the rule model.

	ET-Rule 2	ET-Rule 4	Student’s t test	*p* value
**Clinical variable**				
Tremor duration in years	23.5 ± 17.1	23.8 ± 17.21	*t*(*16*) = −*0*.*039*	*p* = *0*.*969*
Tremor severity^1^	34.7 ± 18.4	33.8 ± 8.38	*t*(*16*) = *0*.*115*	*p* = *0*.*910*

Values are expressed as mean ± standard deviation. ^1^Fahn–Tolosa–Marin Tremor Rating Scale.

## Discussion

During the past decade, voxel-based morphometry (VBM) has been widely used in neurological research. It has proven to be very useful for scientific purposes, although less so for diagnostic purposes. Statistically significant volumetric differences in voxels are not necessarily of diagnostic value, since they might be over-fitted to the studied population and cannot be applied accurately on an individual basis. Volume of regions of interest (ROIs), as well as thickness in the case of cortical areas, avoid the over-fitting effect. However, they do not reflect small deterioration inside the ROIs and might be including other deterioration due to co-morbid or concurrent pathologies. Measuring the roughness (standard deviation of the thickness) of an ROI avoids the need to detect low levels of deterioration, which are typically present at early or intermediate stages of neurodegenerative disease. The deterioration (thinning) of an ROI’s subarea usually corresponds to a high roughness value of that ROI. That might be the reason why the roughness-related features were the best overall classifiers and, consequently, showed the best diagnostic accuracy. However, the measurement of roughness does not solve the problem that one may be measuring other deterioration due to co-morbid or concurrent pathologies that target the same ROI. Given that these other shared pathologies are usually cognitive or psychiatric in nature, roughness analysis can be complemented with correlational neuropsychological assessment to isolate the target pathology for diagnosis.

Typical statistical analysis can reveal ROIs with significant differences between normal and pathological conditions. However, these differences alone cannot be used as diagnostic criteria. Moreover, the pathological profile might be defined by set of features within certain value ranges. In this sense, the data mining approach used in this work has proved to be a plausible method of obtaining diagnostic criteria based on structural MRI by means of a rule model. Such a model only used the roughness of six cortical areas to unequivocally distinguish controls from ET patients. Moreover, the rule model separated ET patients and controls into two different subgroups.

Our finding that six cortical areas allowed for the discrimination between healthy controls and ET patients (present in the rule model) is in agreement with the findings by Benito-León *et al*.^28^, who reported altered functional connectivity within some resting state networks that included these areas. Specifically, some of these areas showed an increased functional connectivity^28^, which suggests that cortical thinning might be caused not only by deterioration, but also by higher functional specialization. In general, reduced functional connectivity is thought to reflect dysfunction of the network, and increased functional connectivity has been interpreted as a compensatory mechanism or reorganization of the network^28^. An increased functional connectivity of a cortical area would imply an increased neural activity in that area and hence higher energy demand, which would ultimate facilitate neuronal damage with the subsequent cortical thinning (i.e., atrophy)^57^. This hypothesis must be investigated further.

Interestingly, the group of control participants belonging to rule 3 presented a roughness of the right inferior parietal area significantly higher than control participants of rule 1. Moreover, this parietal difference was shared with the participants of the two ET rules. Participants in rules 3 and 4 did not present any significant difference in structural or neuropsychological terms. Moreover, they also shared differences with participants in rules 1 and 2. These data point to a special control group, close to the pathological ET group defined by 4. Taking into account that most neuropsychological differences (and correlated structures) between control rule 1 and, control rule 3 and ET rule 4, might be correlated to the difference in years of education, and such a discrepancy was not present between the control rule 1 and ET rule 2, the similarity between ET rule 4 and control rule 3 could plausibly be related to motor features. Consequently, control participants in rule 3 might be susceptible to the development of movement-related disorders.

As noted above, the right inferior parietal cortex was the area that was deteriorated in ET groups 2, 4, and control group 3, with respect to control group 1. A thining of this area with respect to healthy subjects was recently observed in patients with Parkinson’s disease and mild cognitive impairment, who also suffered from postural instability gait disorder (PIGD) phenotype in contrast to tremor dominant (TD) phenotype^58^. This is also in agreement with the findings of Vervoort *et al*.^59^, who found hypo-connectivity between the right inferior parietal area and bilateral M1 and premotor areas in PIGD Parkinson phenotype with respect to TD phenotype. The inferior parietal lobe, particularly in the right hemisphere, has been viewed as a visuomotor interface for object-directed actions^60^, eventually causing difficulties in movement initiation and spatial neglect when deteriorated^61^. This suggests that right inferior parietal changes could be shared by most neuropathologies causing motor-related disorders. Besides, the right inferior parietal area, as defined by the Desikan-Killiany Atlas used in the present study, contains the extrastriate body area (EBA). This area is well-known to respond to visual processing of static and moving human bodies even in the absence of visual feedback from the limb. Moreover, the EBA responds not only during the perception of other people’s body parts, but also during goal-directed movements of the observer’s body parts^62, 63^. Therefore, a deterioration of this area might plausibly cause motor disorders.

In addition, the roughness of the right fusiform area has proven to be a discriminative feature between the groups defined by the rules, and more concretely between the two ET rules. It is well known that the lateral occipitotemporal cortex plays important roles in the perception, understanding and production of action^63^. The fusiform area contains the so-called fusiform body area (FBA), which selectively activates when different body parts are perceived independently from the face fusiform area (FFA)^64–66^. Nevertheless, the FFA has been also shown to be selectively responsive to upper-limb motor actions besides face recognition^67^. Concretely, the right fusiform area showed a greater activation when perceiving whole bodies than when perceiving unconnected body segments^68^. Furthermore, the right FBA has shown an increased functional connectivity with motor area (M1) and supplementary motor area (SMA) during the perception of fluent apparent biological motion (ABM)^69^. This evidence points to an integrative function of the body and its movement in space in the right fusiform area. Consequently, a deterioration of this area could plausibly be related to motor disorders, like the ones present in ET.

Postmortem studies of ET have demonstrated a variety of changes in the cerebellum^70^. While they have not identified gross or microscopic changes in the cerebral cortex, they have not evaluated cortical thickness. By contrast, the current analyses focus on the cerebral cortical thickness and did not include the cerebellum.

Although the sex difference between control and ET conditions is not statistically significant, still more men were included in the ET-group. This difference is not likely to introduce any effect, since this is too small as evidenced by the statistical analysis (*p* = 0.317, Table 1). The literature has evidenced some influence of sex on cortical thickness in healthy elderly, pointing to a greater general thinning in men^71, 72^. However, since the findings of our work are mainly based on cortical roughness (normalized standard deviation of cortical thickness), which means an individual-relative measure, the tentative sex differences have no effect on our results.

The study was not without limitations. First, the sample size was relatively small. However, we could detect significant differences between ET patients and healthy controls even with these smaller numbers. Notwithstanding, it would be important to replicate these findings in a larger sample. Second, the diagnosis of ET was based on clinical criteria and further supported by normal [(123) I]FP-CIT single photon emission computed tomography scan results. None of the ET patients had post-mortem assessments, so that it was not possible to determine whether they had the types of changes that have been reported in ET^70, 73^. Third, the study was designed to distinguish ET cases from controls and we did not include any diseased control groups (e.g., patients with Parkinson’s disease or dystonia). Inclusion of such comparison groups in subsequent studies will allow us to determine whether the findings of this study are unique to ET or whether they extend in part or fully to patients with other movement disorders. Nontheless, the current findings are internally valide and they provide an initial step towards identifying and delineating an ET signature.

In closing, to the authors’ knowledge, the work presented in this paper is the first attempt to use the roughness, as the standard deviation of cortical thickness within a cortical area, for the characterization of a neuropathological condition. In this sense, roughness has shown far more discriminative power than cortical thickness or volume for ET characterization and diagnosis. Moreover, roughness together with data mining techniques provided criteria not only for ET diagnosis, but also for ET subgrouping and characterization from MRI alone. We found that cortical thickness features alone distinguished the two, ET from controls, with 81% diagnostic accuracy. It is possible but remains to be demonstrated whether the combination of this technique, when used in parallel with existing clinical methods (i.e., neurological history and examination), would improve the diagnostic accuracy of the latter. As diagnosis in ET remains a challenge, future work, enrolling a fresh sample of ET cases and controls should explore this promising possibility.

In this study there was evidence in ET patients of deterioration of the right EBA-FBA circuit, which is implicated in guiding goal-directed behavior^74, 75^. This could explain the motor symptoms of ET, mostly seen during action^76, 77^. If this is true, the two implicated cortical areas would be clinical targets of neurostimulation approaches, such as repetitive transcranial magnetic stimulation (rTMS) or transcranial current direct stimulation (tDCS), in order to treat the motor symptoms. Unlike the typical targets (motor area, premotor area and cerebellum), these new targets could eventually provide new insights on long-lasting reduction of motor symptoms^78, 79^.
